# The Correlation between Running Economy and Maximal Oxygen Uptake: Cross-Sectional and Longitudinal Relationships in Highly Trained Distance Runners

**DOI:** 10.1371/journal.pone.0123101

**Published:** 2015-04-07

**Authors:** Andrew J. Shaw, Stephen A. Ingham, Greg Atkinson, Jonathan P. Folland

**Affiliations:** 1 English Institute of Sport, Loughborough University, Loughborough, United Kingdom; 2 School of Sport, Exercise and Health Sciences, Loughborough University, Loughborough, United Kingdom; 3 Health and Social Care Institute, Teesside University, Middlesbrough, United Kingdom; Norwegian University of Science and Technology, NORWAY

## Abstract

A positive relationship between running economy and maximal oxygen uptake (V̇O_2max_) has been postulated in trained athletes, but previous evidence is equivocal and could have been confounded by statistical artefacts. Whether this relationship is preserved in response to running training (changes in running economy and V̇O_2max_) has yet to be explored. This study examined the relationships of (i) running economy and V̇O_2max_ between runners, and (ii) the changes in running economy and V̇O2max that occur within runners in response to habitual training. 168 trained distance runners (males, n = 98, V̇O_2max_ 73.0 ± 6.3 mL∙kg^-1^∙min^-1^; females, n = 70, V̇O_2max_ 65.2 ± 5.9 mL kg^-1^∙min^-1^) performed a discontinuous submaximal running test to determine running economy (kcal∙km^-1^). A continuous incremental treadmill running test to volitional exhaustion was used to determine V̇O_2max_ 54 participants (males, n = 27; females, n = 27) also completed at least one follow up assessment. Partial correlation analysis revealed small positive relationships between running economy and V̇O_2max_ (males r = 0.26, females r = 0.25; P<0.006), in addition to moderate positive relationships between the changes in running economy and V̇O_2max_ in response to habitual training (r = 0.35; P<0.001). In conclusion, the current investigation demonstrates that only a small to moderate relationship exists between running economy and V̇O_2max_ in highly trained distance runners. With >85% of the variance in these parameters unexplained by this relationship, these findings reaffirm that running economy and V̇O_2max_ are primarily determined independently.

## Introduction

Running economy (RE) and maximal oxygen uptake (V̇O_2max_) are two of the primary determinants of endurance running performance [1–4]. The combination of RE and V̇O_2max_, defined as the velocity at V̇O_2max_ (vV̇O_2max_), has been found to account for ~94% of the inter-individual variance in running performance over 16.1 km [5]. Consequently, exceptional values of both RE and V̇O_2max_ are considered requirements for success in elite endurance competitions, and endurance runners strive to improve both parameters through training in order to maximise performance. As the margin of success is extremely small in elite distance running, subtle enhancements in either parameter could result in substantial performance gains. Therefore, understanding the relationship of RE and V̇O_2max_ both between and within individuals is necessary to understand and optimise performance.

Within cohorts of trained [6,7] and elite [8] distance runners, it has been suggested that a superior RE, quantified as the submaximal oxygen uptake, is associated with a lower V̇O_2max_. These findings have been used to postulate that superior economy compensates for a lower V̇O_2max_ in some individual to achieve a similar performance level [3,8,9]. However, these investigations have often been restricted to small sample sizes (<25 participants [3,6,8]), and the validity of their statistical techniques has been questioned due to the expression both variables relative to body mass (i.e. mL∙kg^-1^∙min^-1^); creating a common divisor that is known to produce spurious correlations [10]. Partial correlation analysis would provide an appropriate method to account for the influence of body mass on both variables whilst avoiding statistical artefacts, however this method has yet to be used to examine the relationship between RE and V̇O_2max_. Furthermore, studies have solely employed oxygen cost (O_C_) as a measure of RE, rather than the more valid and comprehensive measurement of energy cost (E_C_; [11]). Thus, whether a genuine association exists between RE and V̇O_2max_ remains unclear from the limited cross-sectional observations to date.

Moreover, the concurrent alterations in RE and V̇O_2max_ that occur within athletes over time with training might further reveal if there is an inherent association between these variables, whilst also informing the optimisation of both variables and thus performance. Previous investigations in well trained athletes have noted enhancements in cycling efficiency following short-term, intensive endurance training, but with no change in V̇O_2max_ evident [9,12,13]. In contrast, a recent investigation reported an association between individual changes in cycling efficiency and V̇O_2max_ in response to endurance training and across a competitive season; despite no change in mean group V̇O_2max_ [14]. These preliminary findings highlight the significance of this relationship for elite endurance athletes, as enhancements in either RE or V̇O_2max_ might only be achievable at the expense of the other variable. However, this previous investigation was limited to measurements of gross efficiency, with no data presented on movement economy. Moreover, analysis of this longitudinal relationship was restricted to observations within small cohorts of athletes, and with responses to run training yet to be explored.

The primary aim of the current investigation was to explore the cross-sectional relationship between V̇O_2max_ and RE, quantified as E_C_ (O_C_ data are also presented for comparative purposes), within a large cohort of highly trained distance runners. The secondary aim was to examine the longitudinal relationship between the changes in V̇O_2max_ and RE occurring within athletes in response to endurance training.

## Materials and Methods

### Overview

The cross-sectional investigation involved retrospective analysis of data from 168 healthy endurance trained athletes with competitive distances ranging from 800m to the marathon (males, n = 98; females, n = 70; Table 1), who undertook testing and monitoring as part of their sport science support from the English Institute of Sport. The following tests were performed after written informed consent was obtained as a part of sports science support provision, with procedures approved by the Internal Review Board of English Institute of Sport. Of the participants assessed, 97 (males, n = 57; females, n = 40) were classed as middle distance runners, defined by a primary competitive distance≤3000m [15], with 71 classed as long distance runners (males, n = 41; females, n = 30). During the season following their final visit, athlete’s best performance times in their primary competitive distance were 89.1±6.1% and 91.2±4.4% of the current British record for males and females, respectively. Data were collected from two laboratories, with all tests conducted as part of athlete support services between November 2004 and April 2013. Participants provided informed consent prior to physiological assessments, in addition to an athlete agreement providing permission for the use of their data in anonymous retrospective analysis. During each visit to the laboratory, participants completed first submaximal and then maximal running assessments (detailed below). Participants wore appropriate clothing (shorts and a vest or t-shirt) and racing shoes, and laboratory conditions were similar throughout all running assessments (temperature 20.6±1.9°C, relative humidity 45.9±9.8%). As differences in RE and V̇O_2max_ have been noted between sexes [16–18], males and females were analysed separately for cross sectional analyses.

**Table 1:** Physiological and anthropometrical characteristics of athletes within the cross sectional and longitudinal investigations.

	**Cross sectional**		**Longitudinal sub-group**	
	**Females**	**Males**	**Females**	**Males**
	(n = 70)	(n = 98)	(n = 27)	(n = 27)
**Age** (yrs)	23±4	23±6	23±5	21±3
**Body mass** (kg)	55.2±4.7	67.1±7.1	55.4±4.3	66.6±6.0
**Stature** (cm)	169±5	179±7	168±4	179±6
**V̇O**_**2max**_ (mL∙kg^-1^∙min^-1^)	65.2±5.9	73.0±6.3	64.5±4.9	73.6±5.9
**vLTP** (km∙h^-1^)	15.5±1.2	17.2±1.3	15.7±1.2	17.6±1.1
**Running economy** (kcal∙kg^-1^∙km^-1^)	1.15±0.09	1.14±0.09	1.13±0.06	1.13±0.07

V̇O_2max_, maximal oxygen uptake; vLTP, velocity at lactate turnpoint.

The longitudinal aspect of the study was based on 54 participants (males, n = 27; females, n = 27) from amongst the larger cohort of 168 runners, that had completed at least one follow up assessment, with a median trial separation of 203 days (range: 37–2567 days) in order to assess within-athlete changes in both RE and V̇O_2max_ over time. The number of repeat assessments in the longitudinal analysis varied between participants, with a median of 3 visits per athlete (range: 2–10 visits), summating to 182 assessments in total. No evidence is currently available regarding sex differences in the concurrent alterations in RE and V̇O_2max_ in response to habitual endurance training, thus data for males and females were combined for longitudinal analysis.

### Protocol

#### Submaximal running assessments

Following a warm-up (~10 min at 10–12 km∙h^-1^), participants completed a discontinuous submaximal incremental test consisting of six to nine stages of 3 minutes continuous running, with increments of 1 km∙h^-1^ on a motorised treadmill of known belt speeds (HP cosmos Saturn, Traunstein, Germany) interspersed by 30 s rest periods for blood sampling. As the speeds assessed were typically between 10.5 km∙h^-1^ and 18 km∙h^-1^, treadmill gradient was maintained at 1% throughout submaximal assessments in order to reflect the energetic cost of outdoor running [19]. This protocol has been shown to reliable measures of running economy when quantified as both E_C_ and O_C_ (typical error ~3%; [20]). Moreover, the controlled laboratory environment enabled assessments of E_C_ whilst avoiding the confounding influence of air resistance that is evident during outdoor running as speed increases [21]. Recent performance times of participants were used to determine an appropriate starting speed to provide ~4 speeds prior to lactate turnpoint (LTP). Increments were continued until blood lactate concentration had risen exponentially, typically defined as an increase in blood lactate of ~2 mmol∙L^-1^ from the previous stage. HR (s610i, Polar, Finland) and pulmonary gas exchange (detailed below) were monitored throughout the test.

#### Maximal running assessments

V̇O_2max_ was determined by a continuous incremental treadmill running ramp test to volitional exhaustion. After a warm-up, participants initially ran at a speed 2 km∙h^-1^ below the final speed of the submaximal test and at a 1% gradient. Each minute, the incline was increased by 1% until volitional exhaustion. The test duration was typically 6–8 minutes.

### Measurements

#### Anthropometry

Prior to exercise on laboratory visits, body mass was measured using digital scales (Seca 700, Seca, Hamburg, Germany) to the nearest 0.1 kg. Stature was recorded to the nearest 1 cm using a stadiometer (Harpenden Stadiometer, Holtain Limited, UK).

#### Pulmonary gas exchange

Breath-by-breath gas exchange data was quantified via an automated open circuit metabolic cart (Oxycon Pro, Carefusion, San Diego, USA). Participants breathed through a low dead-space mask, with air sampled at 60 mL∙min^-1^. Prior to each test, two point calibrations of both gas sensors were completed, using a known gas mixture (16% O_2_, 5% CO_2_) and ambient air. Ventilatory volume was calibrated using a 3 L (±0.4%) syringe. This system has previously been shown to be a valid apparatus for the determination of oxygen consumption (V̇O_2_) and carbon dioxide production (V̇CO_2_) at both low and maximal exercise intensities [22]. As previous data from our laboratory has demonstrated a steady state V̇O_2_, and V̇CO_2_ is achieved within the first 2 minutes of each stage for highly trained endurance runners [20], mean values from breathe-by-breathe measures over the final 60 seconds of each stage were used to quantify V̇O_2_, carbon dioxide production V̇CO_2_, and RER.

#### Blood lactate

A 20μL capillary blood sample was taken from the earlobe for analysis of blood lactate ([La]_b_) (Biosen C-line, EKF diagnostics, Germany). The LTP was identified via the modified Dmax method [23]. LTP was quantified as the point on the third order polynomial curve fitted to the speed-lactate relationship that generated the greatest perpendicular distance to the straight line formed between the stage proceeding an increase in [La]_b_ greater than 0.4 mmol.L^-1^ (lactate threshold) and the final stage. The four stages prior to LTP were identified for each participant, with an average of these four stages used to quantify O_C_ and E_C._

#### Calculation of running economy

V̇O_2_ and V̇CO_2_ during the final minute of each submaximal stage were used to calculate E_C_. Updated nonprotein respiratory quotient equations [24] were used to estimate substrate utilisation (g∙min^-1^) during the monitored period. The energy derived from each substrate was then calculated by multiplying fat and carbohydrate usage by 9.75 kcal and 4.07 kcal, respectively, reflecting the mean energy content of the metabolised substrates during moderate to high intensity exercise [25]. E_C_ was quantified as the sum of these values, expressed in kcal∙km^-1^. V̇O_2_ during the final minute of each submaximal stage was used to determine oxygen cost (O_C_) in mL∙km^-1^ to enable comparisons to previous investigations.

#### Statistical analyses

Data are presented as mean±SD for all dependant variables. Data analysis was conducted using SPSS for windows (v21; IBM Corporation, Armonk, NY). When an individual visited the laboratory for repeated assessments, an average of the assessments was calculated and used for the cross sectional analysis. Pearsons product-moment coefficients were calculated to assess the relationship between body mass and E_C_, O_C_ and V̇O_2max_. As body mass was strongly related to both RE measures (E_C_, O_C_) and V̇O_2max_, partial correlations controlling for body mass, and associated 95% confidence intervals (CI), were used to assess the relationship between absolute V̇O_2max_ and both E_C_ and O_C_. This method removes the influence of body mass on both RE and V̇O_2max_ whilst avoiding spurious correlations created by correlating two variables with a common divisor [26]. For graphical display of these relationships, values of E_C_ and V̇O_2max_ adjusted for body mass for each individual were calculated based on individual residuals. This involved summating the individual’s residual, in comparison to the cohort relationship with body mass (e.g. EC vs body mass), with the group mean for that variable [27]. For the longitudinal analysis, in order to assess any relationships between the changes over time in absolute V̇O_2max_ and the changes in both E_C_ and O_C_ over repeat visits, partial correlation coefficients were calculated using ANCOVA [28]; providing a comprehensive model that accounts variations in both body mass and the number of visits per athlete. Cohen's d effect size descriptors (trivial 0.0–0.1, small 0.1–0.3, moderate 0.3–0.5, large 0.5–0.7, very large 0.7–0.9, nearly perfect 0.9–1, perfect 1) were used to infer correlation magnitude [29]. Significance was accepted at P≤0.05.

## Results

### Participant Characteristics

Participant characteristics are shown in Table 1. The well trained status of the participants was emphasised by the high V̇O_2max_ and vLTP values for both males and females.

### Cross-sectional analysis

**Fig 1:**
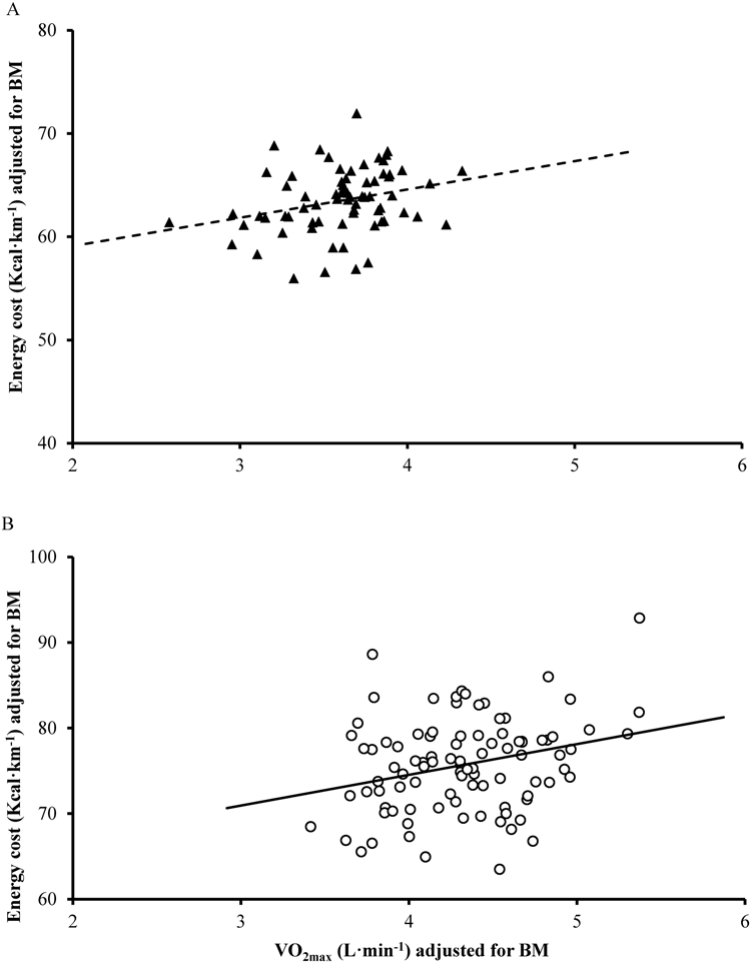
Scatter plot of energy cost (Kcal∙km^-1^) adjusted for body mass (BM) vs V̇O_2max_ (L∙min^-1^) adjusted for BM for both females (A; *n* = 70; r = 0.25; *P* = 0.036) and males (B; *n* = 98; r = 0.26; *P* = 0.009) within the cross-sectional analysis.

Partial correlation analysis controlling for body mass, revealed small positive relationships between E_C_ and V̇O_2max_ (males r = 0.26, CI 0.07–0.44, *P* = 0.009; females r = 0.25, CI 0.02–0.46, *P* = 0.036; Fig 1), and a moderate positive relationship between O_C_ and V̇O_2max_ (males r = 0.33, CI 0.14–0.50, *P* = 0.001; females r = 0.33, CI 0.10–0.52, *P* = 0.006).

### Longitudinal analysis

**Fig 2:**
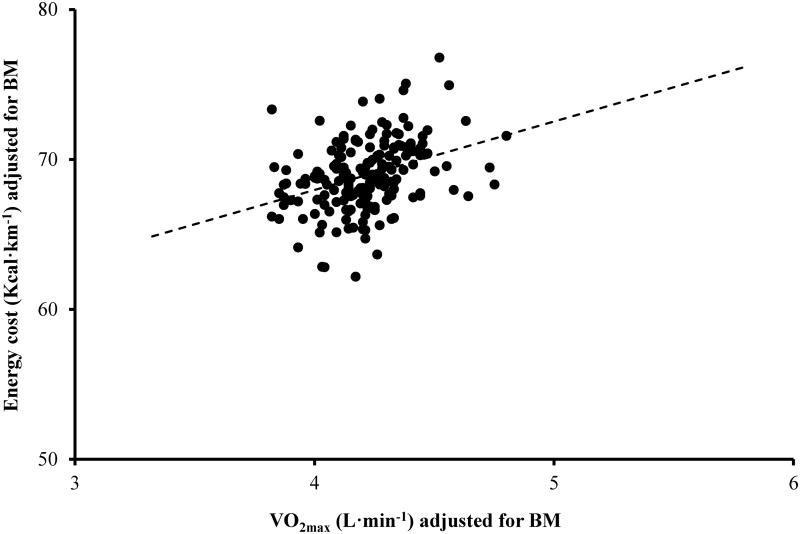
Scatter plot of the changes over time in energy cost (Kcal∙km^-1^) adjusted for body mass (BM) vs the changes over time in V̇O_2max_ (L∙min^-1^) adjusted for BM (r = 0.35; P < 0.001) within the longitudinal analysis.

Partial correlation analysis from ANCOVA revealed moderate positive relationships between the changes in E_C_ and V̇O_2max_ over time (r = 0.35; CI 0.19–0.49, *P* < 0.001; Fig 2), and changes in O_C_ and V̇O_2max_ over time (r = 0.44; CI 0.29–0.57, *P* < 0.001).

## Discussion

The present investigation explored the cross-sectional and longitudinal relationships between RE and V̇O_2max_ in a large cohort of highly trained distance runners. The major contribution of this study to the field is that only a small to moderate association exists between RE and V̇O_2max_ (R^2^ ~ 12%) when body mass is appropriately accounted for. With >85% of the variance in these parameters unexplained by this relationship, these findings reaffirm that RE and V̇O_2max_ are primarily determined independently.

Cross-sectional analysis revealed a small positive between-participant relationships between V̇O_2max_ and the metabolic cost of running, when quantified as both E_C_ (r ~ 0.25) and O_C_ (r ~ 0.33). These results support the findings of Pate et al. [7], who reported a similar relationship (r = 0.29) between submaximal V̇O_2_ and V̇O_2max_ in a similarly large cohort of habitual distance runners. Conversely, a stronger, moderate positive relationship has been reported between submaximal V̇O_2_ and V̇O_2max_ in smaller cohorts of elite distance runners (r = 0.59; [8]) and physically active individuals (r = 0.48; [30]). However, all aforementioned investigations are confounded by statistical artefacts that arise when correlating two variables with common divisors [10,26], and thus should be regarded with caution. Within the current study, spurious correlations between RE and V̇O_2max_ were avoided by removing the influence of body mass with partial correlations, which enabled the true relationship between these variables to be examined. As a lower metabolic cost is reflective of a more economical runner, our findings confirm the existence of a small inverse association between RE and V̇O_2max_ in endurance runners.

The longitudinal analysis of the relationship between the changes in RE and the changes in V̇O_2max_ within participants in response to training has not previously been documented. Supporting the findings from our cross sectional analysis, a moderate positive relationship (r = 0.35) was observed between the changes in E_C_ and V̇O_2max_ over repeated assessments. Moreover, these findings support recent observations from competitive road cyclists that highlighted a similar moderate relationship (r = 0.44) between changes in gross efficiency and V̇O_2max_ across a training season [14].

It has been postulated that variations in lipid oxidation rates between individuals might, in part, explain the relationship between O_C_ and V̇O_2max_ that some previous studies have documented; with a higher V̇O_2max_ facilitating greater lipid oxidation and consequently a greater O_C_ during sub-maximal exercise [7]. Whilst O_C_ may be sensitive to lipid oxidation, the calculation of E_C_ includes the RER and thus is insensitive to differences in substrate metabolism. The influence of substrate metabolism could conceivably explain the marginally stronger relationship observed between O_C_ and V̇O_2max_, than E_C_ and V̇O_2max,_ in both the cross sectional (r ~ 0.33 vs r ~ 0.25) and longitudinal observations (r = 0.44 vs r = 0.35). More importantly, a positive relationship was documented between E_C_ and V̇O_2max_ that is clearly independent of variations in lipid metabolism.

The mechanisms that underpin the small relationship between E_C_ and V̇O_2max_ remain unclear. It has been argued that for athletes of a similar, high performance level, there would be an inevitable relationship between E_C_ and V̇O_2max_ in order to produce a similar velocity at V̇O_2max_ [31]. However, we have found no evidence for this possibility, despite all the participants in this study being highly trained and high performing runners, perhaps in part because of the variable performance ability of the athletes. It is also possible that less economical runners recruit a larger muscle mass (braking, oscillation etc.) and it is conceivable that this could contribute to a higher V̇O_2max_. However there is considerable evidence that V̇O_2max_ during whole body exercise such as running is largely dependent on oxygen delivery rather than utilisation [32], which might question this explanation. Thus, further investigation would be required to identify the factors driving the interdependence of E_C_ and V̇O_2max_.

Though reaching statistical significance, the association between RE and V̇O_2max_ was small. The current study found only ~ 7% (between-participant cross sectional data) and 12% (within-participant longitudinal data) of the variance in RE was explained by V̇O_2max._ This small association likely reflects the distinct nature of these variables and their physiological determinants. V̇O_2max_ is known to be determined by factors such as cardiac output [33], total haemoglobin mass [34], and mitochondrial capacity [1]. Conversely, RE is thought to be closely associated to multiple biomechanical and anthropometrical factors, including effective storage and re-utilisation of elastic energy [35,36], vertical oscillation [37] and ground contact time [38]. As there are few common determinants of both RE and V̇O_2max_, adaptations that lead to enhancements in one of these variables are unlikely to directly influence in the opposing variable.

In conclusion, the current investigation demonstrates that only a small to moderate relationship exists between running economy and V̇O_2max_ in highly trained distance runners. With >85% of the variance in these parameters unexplained by this relationship, these findings reaffirm that running economy and V̇O_2max_ are primarily determined independently.
